# Supplementary material for: First Insights into Ploidy and Genome Size Estimation in Choerospondias axillaris (Roxb.) B.L.Burtt & A.W.Hill (Anacardiaceae) Using Flow Cytometry and Genome Survey Sequencing
Source: Plants (Basel). 2025 Oct 7;14(19):3094. doi: 10.3390/plants14193094 (PMC12527106; doi:10.3390/plants14193094)
Supplement: Supplementary file 1 [file plants-14-03094-s001.zip › plants-3808733-supplementary.pdf]

Supplementary Tables

Table S1. Comparison of flow cytometry results treated with different tissues.

Tissues	Samples	Repeat times	CV%
Leaf emergence	No.22 (QYS13)	3	3.06
Leaf expansion	No.22 (QYS13)	3	2.75
Fully expanded leaf	No.22 (QYS13)	3	4.58
Floral organ	No.22 (QYS13)	3	5.06

Table S2. Comparison of flow cytometry results treated with different lysates.

Nuclear lysis buffer	Samples	Repeat times	CV%
mGb	No.22 (QYS13)	3	4.08
LB01	No.22 (QYS13)	3	5.84
WPB	No.22 (QYS13)	3	2.90

Table S3. Comparison of flow cytometry results treated with different dissociation times

**Dissociation times**	Samples	Repeat times	CV%
0	No.22 (QYS13)	3	5.08
5 min	No.22 (QYS13)	3	3.75
10 min	No.22 (QYS13)	3	5.89

Table S4. 58 samples information of *Choerospondias axillaris*

No.	Code	Original Areas	Longitude	Latitude	internal standard materials	C Value/pg	Genome Size/Mb	Ploidy/n	Coefficient of variation/%
1	YX6	Hengshui Town, Chongyi County, Ganzhou City, Jiangxi Province, China	114.18 E	25.41 N	rice	0.46	451.01	2	6.9
2	QYS17	Yangshuo City, Guangxi Province, China	110.50 E	24.78 N	rice	0.47	461.56	2	5.8
3	YX7	Hengshui Town, Chongyi County, Ganzhou City, Jiangxi Province, China	114.18 E	25.41 N	rice	0.45	441.99	2	5.6
4	YZ6	Hengshui Town, Chongyi County, Ganzhou City, Jiangxi Province, China	114.18 E	25.41 N	rice	0.45	438.73	2	6.7
5	QYS21	Mangshi City, Yunnan Province, China	98.58 E	24.43 N	rice	0.47	461.40	2	6.8
6	YX8	Hengshui Town, Chongyi County, Ganzhou City, Jiangxi Province, China	114.18 E	25.41 N	rice	0.46	448.37	2	6.9
7	YB5	Hengshui Town, Chongyi County, Ganzhou City, Jiangxi Province, China	114.18 E	25.41 N	rice	0.42	410.09	2	6.7
8	ZDU	Hengshui Town, Chongyi County, Ganzhou City, Jiangxi Province, China	114.18 E	25.41 N	rice	0.45	441.47	2	6.9
9	GTXG	Guantian Town, Chongyi County, Ganzhou City, Jiangxi Province, China	114.18 E	25.41 N	rice	0.43	422.32	2	6.7
10	GTLC	Guantian Town, Chongyi County, Ganzhou City, Jiangxi Province, China	114.18 E	25.41 N	rice	0.47	463.96	2	6.2
11	GTLC-2	Guantian Town, Chongyi County, Ganzhou City, Jiangxi Province, China	114.18 E	25.41 N	rice	0.51	494.71	2	6.5
12	GTLC-3	Guantian Town, Chongyi County, Ganzhou City, Jiangxi Province, China	114.18 E	25.41 N	rice	0.49	477.46	2	5.5
13	GTLCDG	Guantian Town, Chongyi County, Ganzhou City, Jiangxi Province, China	114.18 E	25.41 N	rice	0.41	402.85	2	5.4
14	CZ1	Hengshui Town, Chongyi County, Ganzhou City, Jiangxi Province, China	114.18 E	25.41 N	rice	0.50	487.33	2	5.6

15	YSCZ	Hengshui Town, Chongyi County, Ganzhou City, Jiangxi Province, China	114.18 E	25.41 N	rice	0.58	563.89	2	2.4
16	QYS1	Shixing County, Shaoguan City, Guangdong Province, China	114.07 E	24.51 N	rice	0.45	435.92	2	5.0
17	QYS7	Hengshui Town, Chongyi County, Ganzhou City, Jiangxi Province, China	114.18 E	25.41 N	rice	0.44	431.04	2	4.8
18	QYS1-1	Shixing County, Shaoguan City, Guangdong Province, China	114.07 E	24.51 N	rice	0.47	455.38	2	5.5
19	QYS15	Hengshui Town, Chongyi County, Ganzhou City, Jiangxi Province, China	114.18 E	25.41 N	rice	0.46	447.93	2	5.7
20	QYS16	Yiyang City, Hunan Province, China	112.33 E	28.58 N	tomato	0.76	746.64	3	3.4
21	QYS17-3	Yangshuo City, Guangxi Province, China	110.50 E	24.78 N	tomato	0.58	571.01	3	2.9
22	QYS13	Hengshui Town, Chongyi County, Ganzhou City, Jiangxi Province, China	114.18 E	25.41 N	rice	0.89	433.39	2	6.2
23	QYS7-1	Hengshui Town, Chongyi County, Ganzhou City, Jiangxi Province, China	114.18 E	25.41 N	rice	0.47	457.46	2	4.9
24	QYS15-1	Hengshui Town, Chongyi County, Ganzhou City, Jiangxi Province, China	114.18 E	25.41 N	rice	0.45	444.36	2	6.4
25	QYS21-3	Mangshi City, Yunnan Province, China	98.58 E	24.43 N	rice	0.46	454.04	2	5.7
26	YSCZ-1	Hengshui Town, Chongyi County, Ganzhou City, Jiangxi Province, China	114.18 E	25.41 N	rice	0.45	443.48	2	4.9
27	QYS13-1	Hengshui Town, Chongyi County, Ganzhou City, Jiangxi Province, China	114.18 E	25.41 N	rice	0.46	448.88	2	4.3
28	QYS1-2	Shixing County, Shaoguan City, Guangdong Province, China	114.07 E	24.51 N	rice	0.47	458.89	2	5.9
29	QYS18	Ningxiang City, Hunan Province, China	112.53 E	28.28 N	tomato	0.76	744.30	3	3.2
30	QYS15-2	Hengshui Town, Chongyi County, Ganzhou City, Jiangxi Province, China	114.18 E	25.41 N	rice	0.46	453.60	2	4.8
31	QYS19	Xiangtan City, Hunan Province, China	112.92 E	27.83 N	tomato	0.72	700.07	3	4.6
32	QYS17-1	Yangshuo City, Guangxi Province, China	110.50 E	24.78 N	tomato	0.64	622.47	3	4.0
33	QYS19-1	Xiangtan City, Hunan Province, China	112.92 E	27.83 N	tomato	0.71	699.23	3	3.9

34	QYS13-2	Hengshui Town, Chongyi County, Ganzhou City, Jiangxi Province, China	114.18 E	25.41 N	rice	0.46	452.60	2	4.9
35	QYS13-3	Hengshui Town, Chongyi County, Ganzhou City, Jiangxi Province, China	114.18 E	25.41 N	rice	0.50	493.88	2	4.3
36	QYS19-2	Xiangtan City, Hunan Province, China	112.92 E	27.83 N	tomato	0.64	621.76	3	4.0
37	QYS19-3	Xiangtan City, Hunan Province, China	112.92 E	27.83 N	tomato	0.76	746.06	3	5.2
38	QYS20-1	Hengshui Town, Chongyi County, Ganzhou City, Jiangxi Province, China	114.18 E	25.41 N	rice	0.54	532.34	2	4.6
39	QYS23-3	Hengshui Town, Chongyi County, Ganzhou City, Jiangxi Province, China	114.18 E	25.41 N	rice	0.41	403.58	2	6.8
40	QYS21-2	Mangshi City, Yunnan Province, China	98.58 E	24.43 N	rice	0.43	423.82	2	6.5
41	QYS22	Hengshui Town, Chongyi County, Ganzhou City, Jiangxi Province, China	114.18 E	25.41 N	rice	0.44	428.26	2	5.4
42	TX-04	Hengshui Town, Chongyi County, Ganzhou City, Jiangxi Province, China	114.18 E	25.41 N	rice	0.46	451.55	2	4.9
43	QYS1-3	Shixing County, Shaoguan City, Guangdong Province, China	114.07 E	24.51 N	rice	0.45	444.43	2	4.8
44	QYS14	Hengshui Town, Chongyi County, Ganzhou City, Jiangxi Province, China	114.18 E	25.41 N	rice	0.47	455.24	2	4.3
45	QYS18-2	Ningxiang City, Hunan Province, China	112.53 E	28.28 N	tomato	0.62	603.21	3	3.8
46	QYS14-1	Hengshui Town, Chongyi County, Ganzhou City, Jiangxi Province, China	114.18 E	25.41 N	rice	0.47	456.46	2	6.1
47	QYS18-1	Ningxiang City, Hunan Province, China	112.53 E	28.28 N	tomato	0.69	671.77	3	4.3
48	QYS17-2	Yangshuo City, Guangxi Province, China	110.50 E	24.78 N		0.46	449.50	2	6.8
49	QYS16-1	Yiyang City, Hunan Province, China	112.33 E	28.58 N	tomato	0.75	737.04	3	4.6
50	CZ2	Hengshui Town, Chongyi County, Ganzhou City, Jiangxi Province, China	114.18 E	25.41 N	rice	0.46	449.65	2	5.0
51	QYS15-3	Hengshui Town, Chongyi County, Ganzhou City, Jiangxi Province, China	114.18 E	25.41 N	rice	0.46	445.89	2	6.5
52	CZ3	Hengshui Town, Chongyi County, Ganzhou City, Jiangxi Province, China	114.18 E	25.41 N	rice	0.46	449.29	2	6.5

53	QYS14-2	Hengshui Town, Chongyi County, Ganzhou City, Jiangxi Province, China	114.18 E	25.41 N	rice	0.45	440.05	2	5.1
54	QYS14-3	Hengshui Town, Chongyi County, Ganzhou City, Jiangxi Province, China	114.18 E	25.41 N	rice	0.44	432.06	2	5.2
55	QYS14-4	Hengshui Town, Chongyi County, Ganzhou City, Jiangxi Province, China	114.18 E	25.41 N	rice	0.42	411.02	2	5.5
56	QYS21-1	Mangshi City, Yunnan Province, China	98.58 E	24.43 N	rice	0.46	449.50	2	4.1
57	QCM	Hengshui Town, Chongyi County, Ganzhou City, Jiangxi Province, China	114.18 E	25.41 N	rice	0.44	431.17	2	6.6
58	QYS24	Hengshui Town, Chongyi County, Ganzhou City, Jiangxi Province, China	114.18 E	25.41 N	rice	0.44	435.09	2	6.9

Supplementary Figures

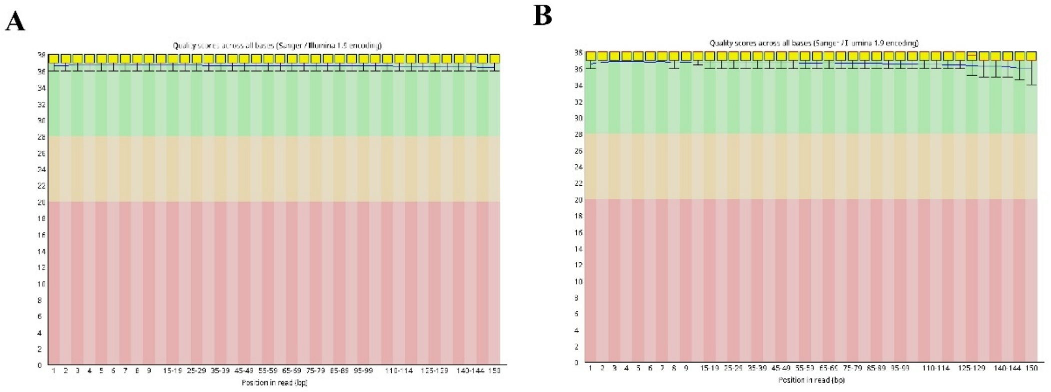

Figure S1. The base quality distribution diagram after sequencing data quality control. (A) Reads 1; (B) Reads 2.

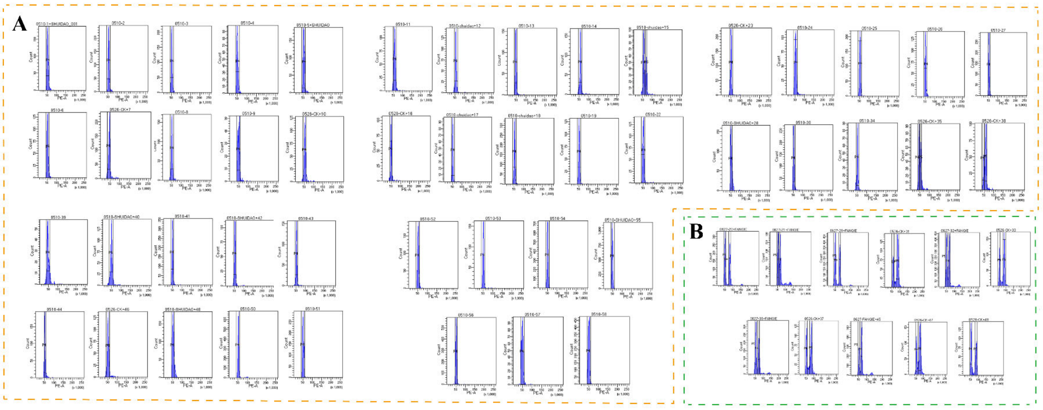

Figure S2. Histogram of 58 *C. axillaris* samples by flow cytometry. (A) 47 diploid *C. axillaris* samples; (B) Histograms of eleven triploid *C. axillaris* by flow cytometry. The X-axis represents the total fluorescence intensity of each detected nucleus. Higher values indicate larger genome sizes or higher ploidy levels. The Y-axis represents the absolute number of cellular events recorded at each fluorescence intensity level. This is a histogram representation where higher peaks indicate more cells with a specific PE signal intensity.
